# Isolated Subclinical Hyperthyrotropinemia in Obese Children: Does Levothyroxine (LT4) Improve Weight Reduction during Combined Behavioral Therapy?

**DOI:** 10.1155/2015/792509

**Published:** 2015-07-02

**Authors:** Pawel Matusik, Aneta Gawlik, Aleksandra Januszek-Trzciakowska, Ewa Malecka-Tendera

**Affiliations:** School of Medicine in Katowice, Department of Pediatrics, Pediatric Endocrinology and Diabetes, Medical University of Silesia, Medykow 16, 40-752 Katowice, Poland

## Abstract

*Objective*. The study aim was to analyze whether anthropometrical parameters and TSH values in obese children with isolated subclinical hypothyroidism (IsHT) treated with levothyroxine (LT4) and weight reduction program differ from those managed by dietary and behavior counselling only. *Material and Methods*. 51 obese children with IsHT, who were treated according to the same weight reduction program, were retrospectively analyzed. They were divided into two groups: Group 1, *n* = 26, and Group 2, *n* = 25, without or with LT4 therapy, respectively. Changes in anthropometrical (delta BMI *z*-score) and hormonal (delta TSH) status were analyzed at the first follow-up visit. *Results*. In both groups significant decrease of TSH and BMI *z*-score values were noted. TSH normalized in 80.9% of children from Group 1 versus 90.5% from Group 2, *p* = NS. Delta BMI *z*-score was insignificantly higher in Group 1 compared to Group 2. Delta TSH was significantly related to initial TSH level in children treated by lifestyle intervention program only. *Conclusions*. In obese children with sHT dietary-behavioral management intervention contributed to reduction of body mass index, irrespective of levothyroxine use. This finding suggests that moderately elevated levels of TSH are a consequence rather than cause of overweight and pharmacological treatment should be avoided.

## 1. Introduction

Childhood obesity epidemic is nowadays the most important challenge for public health system worldwide, mainly in the developed countries [1–3]. Calorie rich diet and sedentary lifestyle are undoubtedly of major importance for weight gain in the population, but the interaction with other factors is far from being elucidated. In recent years there has been increasing focus on the relationship between thyroid function and weight status.

Thyroid function was extensively investigated in obese subjects. Triiodothyronine (T3) promotes thermogenesis and energy expenditure. It also plays a role in regulating food intake and glucose and lipids metabolism. Moreover, thyroid stimulating hormone (TSH), via its receptors in fat tissue, influences differentiation of preadipocytes into adipocytes and promotes expansion of adipose tissue [4–13].

Isolated hyperthyrotropinemia (IsHT) (also known as subclinical hypothyroidism), defined as mildly elevated serum concentration of TSH coexisting with normal peripheral thyroid hormone concentrations, has been commonly reported in obese children. Several studies, mostly cross-sectional and longitudinal, demonstrated a positive correlation between serum levels of TSH and weight status [8–10, 14–22]. Association between obesity and serum TSH level was recently found by Knudsen et al. in Danish population study [10]. As all the subjects investigated had TSH value within the normal limits, the hypothesis was formulated that even slightly elevated TSH may be involved in the pathogenesis of the excessive body weight. On the other hand, studies supporting the concept that obesity by itself is responsible for the changes in thyroid function parameters were published [12, 14, 20, 23–27]. Some of them suggest that production of TSH may be modulated by leptin [24, 25], and direct correlation between serum TSH and leptin concentration has been reported in obese subjects [14, 20, 26]. Another hypothesis of thyroid dysfunction in obesity suggests disturbed negative pituitary feedback, similar to the insulin-resistance phenomenon [12]. Marzullo et al. [27] demonstrated that obesity per se could be also a risk factor for thyroid autoimmunity. However, other studies did not confirm this relationship [8, 9, 11, 13, 15, 22, 28]. The other explanation for the increased TSH level and peripheral thyroid hormones concentration in obese subjects may be an adaptation to increased energy expenditure in order to reduce further weight gain [12].

Progressive fat accumulation is associated with an increase in serum TSH level [4, 10], but this hyperthyrotropinemia was found to be reverted by the weight loss, after both bariatric surgery and hypocaloric diet [19, 20]. Therefore there is continuing debate whether subclinical hypothyroidism in childhood obesity should be treated with thyroid hormone [23].

As there are limited data concerning this issue in pediatric population, the purpose of our study was to evaluate the anthropometrical parameters and TSH values in obese children with isolated subclinical hyperthyrotropinemia treated by levothyroxine and diet plus behavior counselling and to compare the results with children without the pharmacological intervention.

## 2. Material and Methods

The study methodology was based on the retrospective review of charts of the patients who were managed in our obesity outpatient clinic between 2006 and 2011. A total of 51 consecutive series of obese subjects (20 boys, mean age 10.5 ± 3.0 years) with the diagnosis of isolated subclinical hyperthyrotropinemia were identified and included in the study. Medical history, physical examination, and laboratory data were obtained.

Venous blood samples were drawn from antecubital vein in the morning, after the overnight fasting. Concentration of serum free thyroxine (fT4) and thyroid stimulating hormone (TSH) were measured with a chemiluminescent immunometric assay (Siemens, Immulite 2000 Free T4, Immulite 2000 Third Generation TSH, USA). Thyroid peroxidase antibody (anti-TPO Ab) as well as autoantibodies to thyroglobulin (anti-TG Ab) concentrations was determined with enzyme-labeled, chemiluminescent sequential immunometric assay (Siemens, Immulite 2000 anti-TPO Ab, Immulite 2000 Anti-TG Ab, USA). The reference levels for fT4 were 11.5–22.7 pmol/liter (0.8–1.9 ng/dL) and for TSH were 0.4–4.0 mIU/liter. The cut-off points for anti-TG Ab and anti-TPO Ab were below 40 IU/mL and 35 IU/mL, respectively.

The diagnosis of isolated subclinical hyperthyrotropinemia was made when TSH values were elevated (4.0–10.0 mIU/liter) in the presence of normal fT4 concentrations. The main inclusion criterion was negative thyroid autoimmunity (based on anti-TG Ab and anti-TPO Ab evaluation). None of the patients included was taking medication influencing thyroid function (antiepileptics, iodinated drugs, lithium or steroids). They did not have a palpable goiter or other than obesity symptoms commonly associated with hypothyroidism. Detailed anthropometrical analysis was based on the weight and height measurements and body mass index (BMI) calculation, using the standard formula of weight (kg) divided by height (m) squared. BMI *z*-scores were calculated using WHO AnthroPlus, version 1.0.4 (World Health Organization) [29]. Obesity was defined as a BMI at or above the 97th percentile for age and sex, using the WHO charts [29].

The patients were divided into two groups with respect to the levothyroxine (LT4) therapy.

*Group 1*. 26 children (13 boys, mean age 10.0 ± 3.1 years) without LT4 therapy, treated only by dietary and behavioral counseling.

*Group 2*. 25 children (7 boys, mean age 10.9 ± 3.0 years) in which LT4 was introduced before the first visit by the family doctor or by the pediatrician as a consequence of IH diagnosis. This treatment was continued throughout the whole study period.

All children participated in a combined dietary-behavioral-physical activity weight management intervention. The study was performed in non-iodine-deficient area.

### 2.1. Ethical Considerations

The study was approved by the Ethics Committee of Medical University of Silesia. All participants and/or their caregivers gave informed consent. Patients' rights were also approved according to the Helsinki Declaration.

### 2.2. Statistical Analysis

Changes in anthropometric and hormonal data were analyzed at baseline and at the first follow-up visit after starting weight loss therapy. Baseline characteristics of the groups were compared by the two-sample *t*-tests. BMI, BMI *z*-score, and TSH measurements were compared within groups from baseline to the follow-up visit endpoint by the two-sample *t*-tests. Correlations between baseline and endpoint variables were based on linear Pearson's correlation coefficient. All statistical analysis was made by the Statistica 10 PL software and *p* < 0.05 was considered as significant. All results were reported as mean ± standard deviation (SD).

## 3. Results 

**Figure 1:**
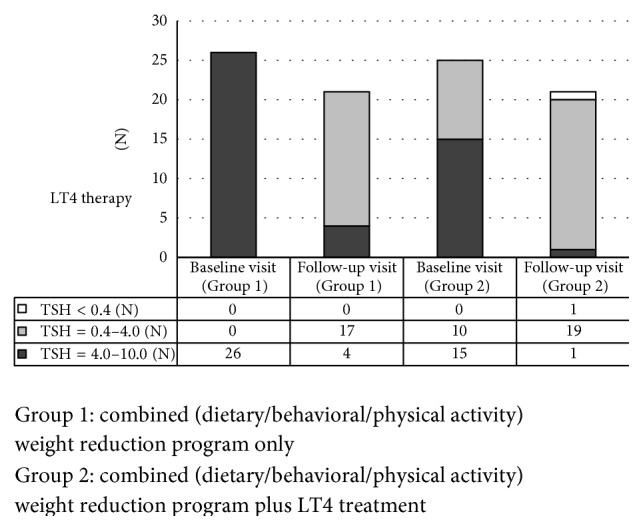
Range of the TSH level at the baseline and at the follow-up visit.

Baseline comparison between groups revealed no significant differences in the mean age and in anthropometrical parameters (Table 1). TSH level in Group 1 was within 4–10 *μ*IU/mL, but 10 (40%) out of 26 children had normal TSH value due to LT4 introduction before the inclusion in the study (Figure 1). This treatment resulted in lower mean baseline TSH level in Group 2 than in Group 1, but the difference was on the border of significance (Table 1). Prior to the baseline visit LT4 therapy duration was from 4 to 8 months and LT4 dose varied between 0.32 and 1.59 *μ*g/kg/day (25–75 *μ*g/day). Mean time from the study starting point to the follow-up visit was 3.35 ± 2.43 versus 5.28 ± 4.3 months for Group 1 and Group 2, respectively (*p* > 0.05). Nutritional status assessed by BMI and BMI *z*-score did not differ significantly between the groups. However, TSH level was significantly lower in the LT4 treated group at the follow-up visit (Table 1).

The same number of patients (*n* = 21) out of each group presented for the follow-up visit. Despite the lifestyle intervention program two patients from Group 1 and four from Group 2 gained weight. The vast majority of patients from Group 1 (*n* = 17 (80.9%)) normalized their TSH level at the follow-up visit and none of them increased their TSH level over 10 *μ*IU/mL. Nineteen children (90.5%) from Group 2 had TSH levels within normal ranges. However in one child a transition to the subclinical hyperthyroidism was noted, so LT4 therapy was discontinued. This patient had the largest BMI decrease from the entire group (delta BMI *z*-score = −0.91 SD).

**Figure 2:**
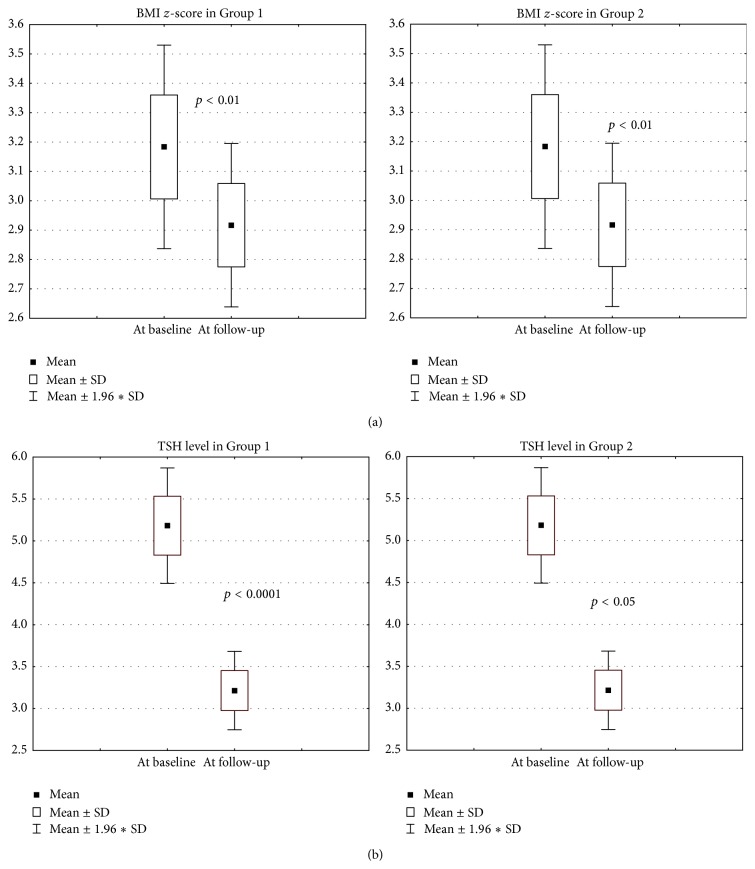
Change of BMI *z*-score at the follow-up visit in both groups. (b) Change of TSH level at the follow-up visit in both groups.

**Figure 3:**
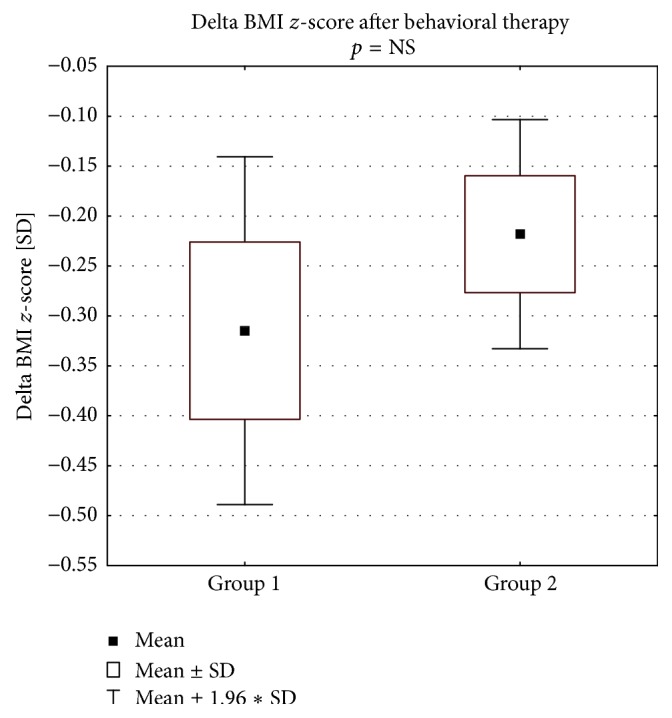
Change of the nutritional status (by delta BMI *z*-score) at the follow-up visit.

TSH concentration changes at the end of the study period are presented in Figure 1. At the follow-up visit, as compared to the baseline, TSH and BMI *z*-score values in Group 1 as well as in Group 2 (Figures 2(a) and 2(b)) were significantly lower. The degree of weight reduction (expressed as delta BMI *z*-score) was higher in Group 1 than in Group 2; however this difference did not reach statistical significance (Figure 3).

**Figure 4:**
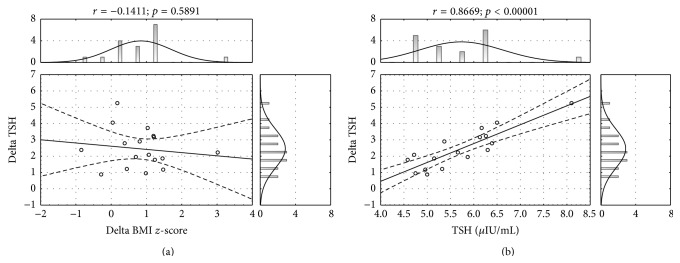
Relationship of change in TSH level (delta TSH) with respect to the nutritional status improvement (delta BMI *z*-score) (a) and with respect to the initial TSH level (b), in the group of obese children with no LT4 treatment (Group 1).

There was no significant correlation between change of TSH level and delta BMI *z*-score in pharmacologically untreated children (Figure 4(a)). However, final decrease of TSH level was significantly related to the initial TSH value in children treated by lifestyle intervention program only (Figure 4(b)).

## 4. Discussion

Our study demonstrates that in obese children with IsHT levothyroxine treatment does not improve the results of weight reducing therapy based on diet and physical activity modification. It also confirms the results of other studies demonstrating that weight reduction may decrease TSH serum level in obese children with IsHT [20, 21] raising the question of the necessity to treat them with levothyroxine.

Isolated hyperthyrotropinemia is quite common in adults and in adolescents, with the worldwide prevalence ranging from 4 to 10% in large general population screening surveys [30] and from 7 to 26% in studies performed in the elderly [31]. In general pediatric population the IsHT prevalence seems to be distinctly lower [32]; however in obese subjects it is estimated to be 7–23% [8–10, 14–22]. The evidence for positive effect of levothyroxine supplementation in children with subclinical hypothyroidism is very poor, although this does not necessarily imply the lack of benefit [33]. Treatment is more likely to be advisable in patients with Hashimoto disease, since progression from IH to overt hypothyroidism is more probable [34]. In the present study, however, an underlying Hashimoto thyroiditis has been preliminarily excluded according to our recruitment criteria.

To our knowledge, in the pediatric setting, there are no well-controlled, longitudinal studies comparing the outcomes of levothyroxine treatment in children with elevated TSH levels versus those receiving placebo or no therapy. Most of them are retrospective, focused on the effect of LT4 treatment on growth velocity [35, 36], thyroid volume [37, 38], or cognitive function [39] and only one is analyzing BMI SDS changes [35]. Two of them investigated the effect of levothyroxine therapy in autoimmune thyroiditis [37, 38]. In one longitudinal nonrandomized study conducted by Wasniewska et al. [35] TSH values in children with subclinical hypothyroidism were evaluated 3 months after therapy withdrawal and compared with untreated control group. According to their results treatment did not modify posttherapy outcome of hyperthyrotropinemia and did not prevent the risk of further TSH increase after treatment withdrawal. Although this study was not conducted in obese children, no significant difference between the two groups was found in terms of BMI SDS, both at the study entry and after 24 months. In spite of the apparent increase of BMI SDS observed in treated children during the 2-year follow-up, the percentage of overweight subjects remained stable in this group. In 42 children who normalized TSH after LT4 therapy withdrawal, there was no difference in BMI evolution during the entire treatment period.

Our study is one of the first reporting the results of LT4 treatment versus no treatment in two comparable groups of obese children with IsHT. All children participated in a combined weight intervention protocol. At follow-up visit we did not find differences in BMI as well as in BMI *z*-score between the children treated and untreated with LT4. Moreover, the tendency to the greater weight loss (expressed as change in BMI *z*-score) was higher in children without the pharmacological intervention.

These results are in concordance with the outcomes of the study conducted by Eliakim et al. [9]. The authors analyzed data of 196 obese children and adolescents and 41 (20.9%) of them were diagnosed with subclinical hypothyroidism. All the subjects with subclinical hypothyroidism were enrolled into weight reduction program and fifteen were also treated with LT4 for 6 months. Hormonal treatment had no significant effect on body weight, linear growth, and lipid profile, except for causing a greater decrease in triglyceride levels. TSH concentrations returned to normal ranges in majority of children with hyperthyrotropinemia who participated in the intervention program irrespective of levothyroxine treatment. As the results of this study are in agreement with our findings, the authors also concluded it with the statement that thyroid hormone substitution in obese children is in most cases unnecessary.

Obese children who reduced their overweight as a result of lifestyle intervention program only decreased significantly their TSH level. This finding is in agreement with several other studies [17, 19, 20]. However Reinehr and Andler in one of their previous studies [18] demonstrated a decrease in peripheral thyroid hormones as opposed to TSH.

In our study no correlation between the change of TSH level and BMI *z*-score was found which is in accordance with other studies results [14, 17]. It is worth highlighting that finally 80% of children from Group 1 versus 90% from Group 2 had TSH within normal ranges. So irrespective of levothyroxine treatment one can expect weight reduction and thyroid biochemical marker improvement, as it was previously suggested by other authors [9, 35].

The range of the dose of levothyroxine applied in our patients was very wide (0.32–1.59 *μ*g/kg/day). As compared to the starting dose of 2 *μ*g/kg per day in other studies [35, 36] it was also rather low, even though it resulted in the normalization of TSH values. Moreover, in one patient it caused the transition to subclinical hyperthyroidism and discontinuation of the therapy.

Basal metabolic rate, total and sleep energy expenditure are positively correlated with the serum T3 or fT3 concentration. In our study we did not analyze changes in FT3 and FT4. In obesity FT3/FT4 ratio was reported to be positively associated with body mass index [23]. This finding suggests a high conversion of T4 to T3 in patients with central obesity due to increased deiodinase activity as a compensatory mechanism for fat accumulation to improve energy expenditure [40]. Hence, changes in thyroid hormone concentrations may be regarded as a consequence rather than a cause of obesity [4, 23].

In the recently published study Reinehr et al. analyzed thyroid hormones in female adolescents with obesity and anorexia nervosa before and after normalization of weight [20]. They were able to demonstrate that obese adolescent females had moderately increased TSH and fT3 concentrations, while girls with anorexia nervosa had slightly decreased fT3 and TSH levels compared to healthy normal-weight girls of the same age. These biochemical markers of thyroid function normalized in obese girls who reduced weight and in girls with anorexia who gained weight, suggesting that the alterations of TSH and peripheral thyroid hormones are reversibly related to weight status. Nevertheless, these findings have not been unanimously agreed upon. Another German group, analyzing data of 290 obese children, found no significant relationship between changes in TSH level and changes in BMI standard deviation score (BMI *z*-score); however a significant correlation was documented between the decrease in TSH level and the decrease in waist circumference [22]. On the other hand Aeberli et al. [14] in prospective study demonstrated that changes in TSH did not correlate with changes in body weight or body composition. Marras et al. reported that, after 6 months of lifestyle intervention, thyroid hormones concentration normalized in only 27 of the 41 children with decreased SDS-BMI. In addition, no correlation was found between the degree of weight loss and reduction of serum TSH concentration. Nevertheless, the author concluded that moderate weight loss frequently restores thyroid abnormalities [17].

We found that follow-up change in TSH level was dependent on the baseline value: the higher the baseline level the more evident the final decrease of TSH. It is difficult to explain this finding and it is controversial to the results demonstrated by Wasniewska et al. [35].

We recognize the limitations of our study such as short time of observation and different duration of therapy as well as discrepancies in levothyroxine dosing. Nevertheless, limited data in the literature concerning this issue make our results noteworthy.

In conclusion, according to the present study, in obese children and adolescents with isolated hyperthyrotropinemia combined dietary-behavioral intervention contributed to the reduction of body mass index irrespective of levothyroxine treatment. As in vast majority of children untreated with levothyroxine who reduced their overweight TSH concentration normalized, IsHT seems to be a consequence rather than cause of the excessive body weight, and therefore pharmacological treatment should be avoided.

## Figures and Tables

**Table 1:** Characteristics of groups.

	Group 1	Group 2	*p* value
	(*n* = 26)	(*n* = 25)	
At baseline visit			
Age (years)	10.0 ± 3.1	10.9 ± 3.0	0.324
Sex (M : F)	13 : 13	7 : 18	—
Weight (kg)	63.4 ± 23.0	62.4 ± 20.7	0.881
Height (cm)	147.1 ± 18.9	147.9 ± 14.9	0.870
BMI (kg/m^2^)	28.2 ± 4.2	27.8 ± 5.6	0.774
BMI *z*-score (SD)	3.4 ± 1.11	2.9 ± 1.04	0.087
TSH (*μ*IU/mL)	5.45 ± 0.86	4.45 ± 2.37	0.052

At follow-up visit			
BMI (kg/m^2^)	27.9 ± 4.2	27.4 ± 4.6	0.719
BMI *z*-score (SD)	3.17 ± 0.86	2.66 ± 0.92	0.072
TSH (*μ*IU/mL)	3.59 ± 1.01	2.59 ± 2.63	0.039

Data are expressed as mean ± standard deviation.

Group 1: combined (dietary/behavioral/physical activity) weight reduction program only.

Group 2: combined (dietary/behavioral/physical activity) weight reduction program plus LT4 treatment.
